# Cardiovascular Protection of Hydroxychloroquine in Patients with Sjögren’s Syndrome

**DOI:** 10.3390/jcm9113469

**Published:** 2020-10-28

**Authors:** Deng-Ho Yang, Yu-Hsun Wang, Lung-Fa Pan, James Cheng-Chung Wei

**Affiliations:** 1 Division of Rheumatology/Immunology/Allergy, Department of Internal Medicine, Taichung Armed-Forces General Hospital, Taichung 411, Taiwan; deng6263@ms71.hinet.net (D.-H.Y.); lung-fa@803.org.tw (L.-F.P.); 2 Department of Medical Laboratory Science and Biotechnology, Central Taiwan University of Science and Technology, Taichung 406, Taiwan; 3 Division of Rheumatology/Immunology/Allergy, Department of Internal Medicine, Tri-Service General Hospital, National Defense Medical Center, Taipei 114, Taiwan; 4 Department of Medical Research, Chung Shan Medical University Hospital, Taichung 402, Taiwan; cshe731@csh.org.tw; 5 Graduate Institute of Radiological Science, Central Taiwan University of Science and Technology, Taichung 406, Taiwan; 6 Department of Rheumatology & Immunology, Chung Shan Medical University Hospital, Taichung 402, Taiwan; 7 Institute of Medicine, Chung Shan Medical University, Graduate Institute of Integrated Medicine, China Medical University, Taichung 402, Taiwan

**Keywords:** Sjögren’s syndrome, hydroxychloroquine, inflammation, coronary artery disease, cardiovascular event, autoimmune

## Abstract

Sjögren’s syndrome (SS) is a chronic systemic inflammation disease with clinical presentation of dry eye, dry mouth, and polyarthralgia. Active inflammation is associated with an increased risk of associated arterial stiffness or subclinical atherosclerosis-related cardiovascular events. We used the longitudinal health insurance database of Taiwan, which includes one million participants, to evaluate the relationship between the clinical medication of hydroxychloroquine (HCQ) and the development of coronary artery disease (CAD). In total, 1674 patients with SS receiving HCQ medication were included after exclusion for previous CAD. Altogether, 1142 SS patients were included for evaluation after follow-up for more than one year. After adjusting for age, gender, medications, and chronic comorbidities, a significantly decreased hazard ratio (HR) for developing CAD was found among SS patients with higher medication possession ratio (MPR) of HCQ (HR = 0.49, 95% confidence interval, CI: 0.26–0.94) when compared with low MPR of HCQ. A low HR for CAD was observed in SS patients with a high cumulative dose of at least 100,267 mg of HCQ (HR = 0.25, 95% CI: 0.09–0.66). Long-term HCQ therapy may decrease the HR of CAD in SS patients. The significant cardiovascular protective effect of HCQ therapy was observed in our study.

## 1. Introduction

Sjögren’s syndrome (SS) is a systemic inflammatory disease with immunologic abnormalities characterized by different clinical manifestations, including dry eye, dry mouth, arthralgia, fatigue, leucopenia, glomerulonephritis, and peripheral neuropathy [1]. The two major features are ocular and oral glandular involvement with impaired function of glands. Active lymphocytic infiltration of the salivary and lacrimal glands may be observed from pathologic findings in patients with SS. In the local pathogenesis of different glands, proliferation of B cells, activation of dendritic cells, and autoreactive T helper 1 and T helper 17 cells develop among the salivary epithelium [2]. Throughout the systemic inflammation, the presentation of proinflammatory cytokines, including interleukin-17 and interferon-γ, are activated [1]. Traditional risk factors for the progression of coronary artery disease (CAD) include age, sex, hypertension, smoking, diabetes, obesity, and hyperlipidemia. Systemic inflammation may also serve as one risk for the development of CAD. Increased risks of CAD have been described in previous studies in other systemic autoimmune diseases, such as rheumatoid arthritis and systemic lupus erythematosus. SS is also a chronic autoimmune inflammatory disease, which is highly associated with atherosclerosis [3]. Cerebrovascular events and CAD are more common in patients with primary SS when compared with the general population [4]. An increased frequency of high carotid intima-media thickness and arterial stiffness is found in patients with primary SS [5]. Patients with primary SS are at risk of developing subclinical atherosclerosis and arterial stiffness. Rates of anxiety, along with impaired sleep, are higher among patients with SS, and impaired sleep may create a significantly higher risk of subclinical atherosclerosis [6]. Hydroxychloroquine (HCQ) is an antimalarial agent that has been used for the treatment of different autoimmune diseases, including rheumatoid arthritis, systemic lupus erythematosus, and SS. The clinical application of HCQ for autoimmune diseases is to control disease activity by immunomodulation. HCQ was one of the first drugs applied in the treatment of SS that resulted in significant improvement of arthritis or fatigue [7]. From a prospective and double-blind study, the medication of HCQ did not improve clinical symptoms of SS significantly without decreasing autoantibodies and high immunoglobulin G levels [8]. However, the duration of this study was 24 weeks of treatment, and further studies are needed to evaluate the longer-term effects of HCQ. Previous studies indicated that the effects of anti-arthrosclerosis medications or those that prevent cardiovascular events (excluding immunosuppressants) may be seen in patients with rheumatoid arthritis and systemic lupus erythematosus [9,10,11,12]. Significant decreasing risk of cardiovascular events is observed when patients with autoimmune diseases receive HCQ therapy. However, the level of cardiovascular protection is still not clear in SS patients who use HCQ on a long-term basis. In our study, we evaluated the incidence of CAD in SS patients who are using HCQ long-term.

## 2. Materials and Methods

### 2.1. Data Source

We conducted a retrospective cohort study by using the National Health Insurance Research Database (NHIRD), which enrolls almost 99% of the population in Taiwan. The dataset consists of all claims in health care, including medical visits, emergency care, and hospitalization. One million participants were sampled from the 23 million beneficiaries, and data were collected from 2000 to 2012. The data in the NHIRD were encrypted, and this study was approved by the Institutional Review Board of Chung Shan Medical University Hospital.

### 2.2. Study Population and Exposure

The study population consisted of patients newly diagnosed with SS (ICD-9-CM = 710.2), aged 20 years or more, from 2000 to 2012. Administration of HCQ within one year of diagnosis was necessary for inclusion. A total of 1674 SS patients treated with HCQ were enrolled. To confirm new-onset CAD, we excluded those with CAD diagnosed before the index date. Therefore, 1142 SS patients were included after exclusion for previous CAD. The index date was the first date of usage of HCQ. To perform the adherence of HCQ, we used the medication possession ratio (MPR) that was from 0 to 1. The closer to 1, the higher compliance the patients had with the medication. The MPR was the total HCQ use days from the SS diagnosis to the index date divided by the time of one year. The baseline characteristics were age, gender, hypertension (ICD-9-CM = 401–405), hyperlipidemia (ICD-9-CM = 272.0–272.4), diabetes (ICD-9-CM = 250), chronic obstructive pulmonary disease (ICD-9-CM = 491, 492, 496), and stroke (ICD-9-CM = 430–438). Those comorbidities were defined before the index date within one year and more than three outpatient visits or a hospitalization. Corticosteroids and non-steroidal anti-inflammatory drugs (NSAIDs) during the study period were included and defined by a minimum usage of ≥30 days.

### 2.3. Outcome

**Figure 1:**
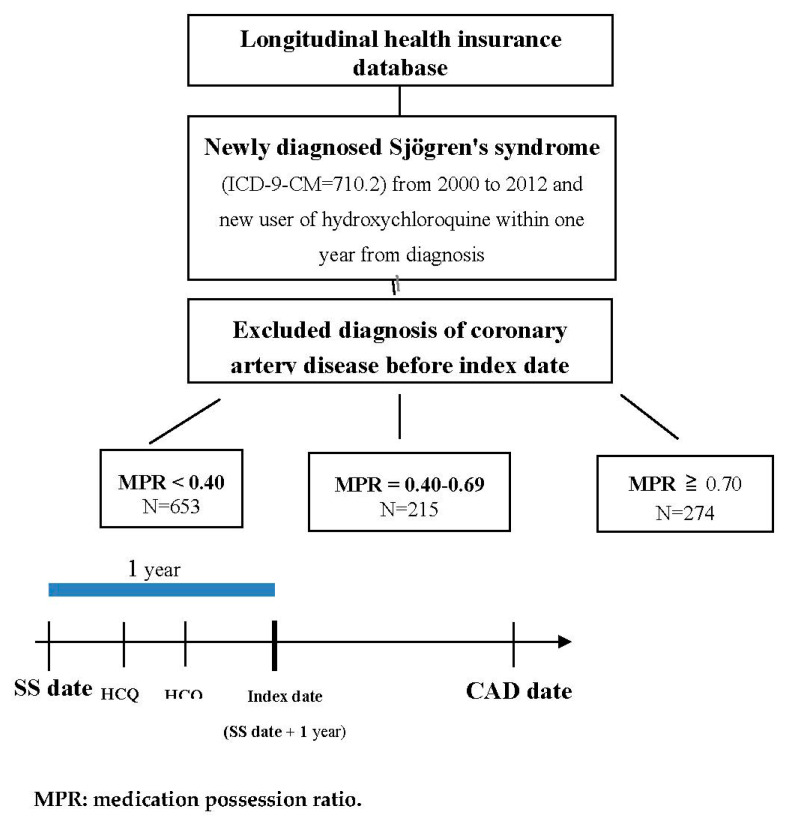
The study flow chart to identify these Sjögren’s syndrome (SS) patients.

The outcome variable was defined as a diagnosis of CAD (ICD-9-CM = 410–414). Additionally, more than three outpatient visits or a hospitalization due to CAD were required for inclusion. Patients were followed up with until the occurrence of CAD, the date of 31 December 2013 was reached, or withdrawal from the national insurance system, whichever occurred first. The total number of SS patients was 1142 after follow-up for more than one year. The study flow chart to identify these SS patients is shown in Figure 1.

### 2.4. Statistical Analysis

The Kaplan–Meier analysis was used to estimate the cumulative incidence of CAD across the HCQ groups, and the log-rank test was used to evaluate the significance. The Cox proportional hazard model was used to estimate the hazard ratio (HR) of CAD in relation to HCQ medication and was adjusted for potential confounding variables. We used statistical software SPSS, version 18.0 (SPSS Inc., Chicago, IL, USA). A *p* value less than 0.05 was considered to indicate significance.

## 3. Results

### 3.1. Study Population

The 1142 SS patients were divided into three groups by medication possession ratio (MPR) to evaluate the drug adherence of HCQ. The three groups were shown as: MPR < 0.40 (N = 653), MPR = 0.40–0.69 (N = 215) and MPR ≥ 0.70 (N = 274). Lower prevalence of hyperlipidemia was observed in the MPR ≥ 0.70 group when compared with the MPR < 0.40 group (3.3% vs. 7.8%, *p* = 0.022). Other chronic comorbidities, including hypertension, diabetes, chronic obstructive pulmonary disease, and stroke, had no significant difference between the three MPR groups. A higher proportion of corticosteroid medication was found in the MPR ≥ 0.70 group when compared with the MPR < 0.40 group (58% vs. 28.8%, *p* < 0.001). The data are shown in Table 1.

### 3.2. A Higher Incidence of CAD Developed in the SS Patients with a Lower MPR (<0.40) of HCQ, and There Was Significant Decreasing Cumulative Incidence of CAD in the SS Patients with a Higher MPR (≥0.70) of HCQ

**Figure 2:**
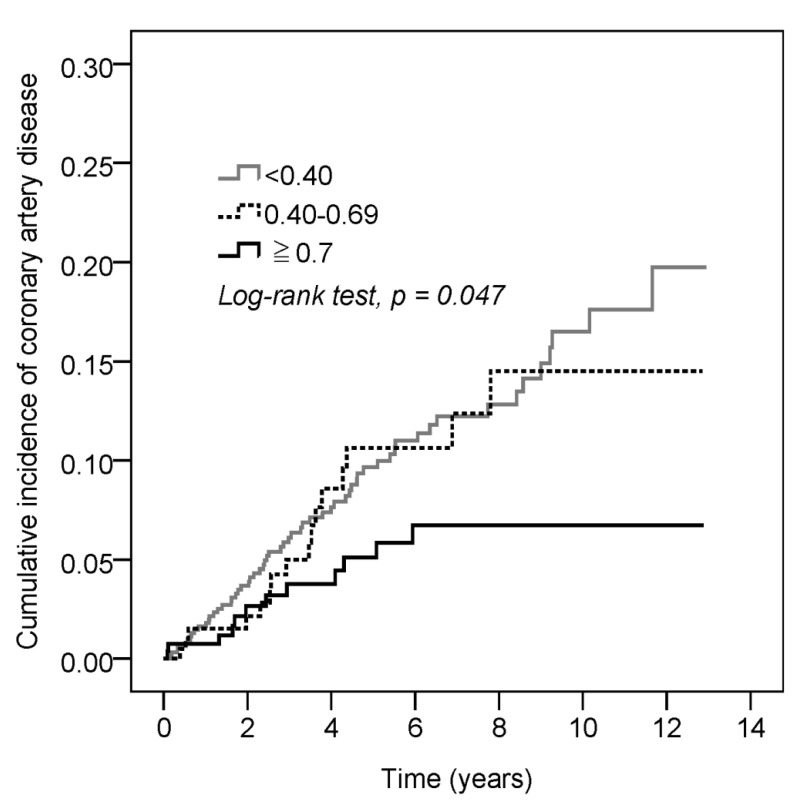
The cumulative incidence of CAD in the three groups with significant decreasing cumulative incidence of CAD in the group with higher MPR (≥0.70) of HCQ.

Significantly lower HR of CAD was observed in the patients with higher MPR (≥0.70) of HCQ when compared with the patients with lower MPR (<0.40) of HCQ (HR = 0.49, 95% CI: 0.26–0.94), after adjusting for age, comorbidities, and gender (Table 2). At the same time, traditional risk factors for CAD, including old age, hypertension, and hyperlipidemia, with higher HR were also observed (age ≥ 65: HR = 4.69, 95% CI: 1.99–11.03; hypertension: HR = 1.98, 95% CI: 1.17–3.36; hyperlipidemia: HR = 2.27, 95% CI: 1.18–4.38). In our study, SS patients with a comorbidity of chronic obstructive pulmonary disease may have higher HR for incidence of CAD (HR = 3.57, 95% CI: 1.51–8.46). There was no significant increasing risk of CAD with the use of corticosteroids or NSAIDs. The cumulative incidence of CAD was significantly lower in the SS patients with a higher MPR (≥0.70) of HCQ (Figure 2).

### 3.3. Significant Cardiovascular Protection of HCQ in the 40–64 Age Group among the Patients with SS

When the age of SS patients was less than 40 years or more than 65 years, there was no significantly different incidence of CAD between a higher and lower MPR of HCQ. However, a different condition was observed in the SS patients aged 40–64. In this group, a significantly lower HR of CAD developed in the SS patients with a higher MPR (≥0.70) of HCQ when compared with a lower MPR (<0.40) of HCQ (HR = 0.28, 95% CI: 0.12–0.68). Among the female SS patients, a lower HR of CAD was also found in the SS patients with a higher MPR (≥0.70) of HCQ when compared with a lower MPR (<0.40) of HCQ (HR = 0.36, 95% CI: 0.16–0.77). However, there was no significant cardiovascular protection between a higher and a lower MPR of HCQ among the male SS patients. The evaluation of CAD attack in the patients with SS by different age and gender is shown in Table 3.

## 4. Discussion

In our study, a higher incidence of CAD was found in the SS patients with a lower MPR of HCQ, and the incidence was decreased in the SS patients with a higher MPR of HCQ. The cumulative incidence of CAD was significantly lower in the SS patients with a higher MPR of HCQ when compared with SS patients with a lower MPR of HCQ. The risk factors for CAD, including old age, hypertension, hyperlipidemia, and COPD, were observed in our data.

SS is a chronic systemic autoimmune disease, and different extraglandular features may develop during the disease progression. Areas involved include skin, thyroid gland, lungs, and the musculoskeletal systems. The cardiovascular system is one of the major areas for extraglandular manifestations. Patients with primary SS have a higher cardiovascular involvement rate than that of the controls [4]. Subclinical atherosclerosis, myocardial infarction, venous thromboembolism, hypertension, and hyperlipidemia can develop in the cardiovascular system. Cerebrovascular events and myocardial infarction are more common among the primary SS patients [13]. The presentation of extraglandular manifestations is associated with an increased comorbidity burden, and cardiovascular disease is one of major associations [14]. Persistent endothelial dysfunction-related subclinical atherosclerosis may be found in SS patients with elevation of endothelial microparticles and endothelial progenitor cells [15]. The prevalence of valvular regurgitation, pericardial effusion, and pulmonary hypertension is higher in SS patients with correlations of immunologic abnormality [16]. Our study showed that hyperlipidemia, hypertension, and COPD may serve as higher risks for the progression of CAD. Among patients with SS, a significantly higher prevalence of hypertension or hyperlipidemia is observed when compared with that in the general population [13,17]. During the disease course of SS, different medications, including glucocorticoids, NSAIDs, and immunosuppressive agents may be used to control the disease activity. NSAIDs may be associated with an increased risk of cardiovascular events in patients with SS [18]. However, in our study, there was no significant difference when using NSAIDs or glucocorticoids. Higher glucocorticoid exposure is associated with CAD risk increase, but minimizing glucocorticoid use may improve CV outcomes in patients with rheumatoid arthritis due to under control of disease activity [19]. The patients with SS usually receive a lower dosage of glucocorticoid when compared with rheumatoid arthritis or systemic lupus erythematosus patients. Adequate control of SS patients’ related systemic inflammations by glucocorticoids may explain this phenomenon. Rheumatoid arthritis is another systemic inflammation autoimmune disease and chronic inflammation may serve as one of the risk factors for the progression of CVD. Aggressive control of the disease activity of rheumatoid arthritis can be achieved by adequate medication with methotreaxate, and the systemic inflammation is improved. Methotrexate and biologics use was associated with CVD risk reduction in an observational study [19]. Therefore, high risk of cardiovascular events in SS patients may be associated with long disease duration, more severe disease activity with active immunologic or clinical features, and the agent to control disease activity.

HCQ is a traditional immunosuppressive drug used for different autoimmune diseases. Except for the effects of immunomodulation, multiple efficacies, including protection against the progression of diabetes, thrombotic events, and dyslipidemia, have been reported in previous studies [4,20,21,22]. A lower percentage of hyperlipidemia was observed in the MPR ≥ 0.70 group when compared with the MPR < 0.40 group (3.3% vs. 7.8%, *p* = 0.022) in our study (Table 1). Previous studies have indicated that a longer duration of HCQ use is associated with better lipid profiles in patients with rheumatoid arthritis [23]. SS is a chronic inflammation-related autoimmune disease, and a higher prevalence of subclinical atherosclerosis may be associated with this autoimmune dysregulation-related systemic inflammation [3]. Endothelial dysfunction and damage-related impaired vasodilatation play major roles in atherosclerosis pathogenesis. HCQ may prevent endothelial dysfunction in the mouse model of antiphospholipid syndrome [24]. HCQ can modulate the production of proinflammatory cytokines, the activation of toll-like receptor and leukocyte generation of reactive oxygen species [25]. These inflammatory-related progressions may serve as important in the development of atherosclerosis in cardiovascular diseases. Adequate control of disease activity and systemic inflammation can improve the incidence of subclinical atherosclerosis. In our study, relatively good drug adherence to HCQ and corticosteroids was found in the MPR ≥ 0.70 group. SS-related systemic inflammation may be regulated by these immunosuppressive therapies. Therefore, a significant decreasing cumulative incidence of CAD was found in the higher MPR (≥0.70) of the HCQ group (Figure 2). Male gender is one of the risk factors for developing CAD, although our study showed that there was no significant difference in incidence between males and females. This condition may be due to a lower percentage of males (13%) in our study. Therefore, significantly lower incidence of CAD was found in the group of female SS patients with higher MPR (≥0.70) of HCQ when compared with the lower MPR (<0.40) of HCQ group (HR = 0.36, 95% CI: 0.16–0.77). This significant difference could not be observed among the male SS patients and this condition may be due to the small number of males in our study. However, the clinical symptoms of SS, including dry eye, dry mouth and arthralgia, are usually found among the patients with IgG4-related disease (IgG4-RD) [26]. Abnormal serum studies including hypergammaglobulinemia, hypocomplementemia, and autoantibodies are also observed. IgG4-RD typically affects middle aged and older men [27]. In clinical practice, this is difficult to distinguish from SS and IgG4-RD. This may be another reason why there was no significant difference in elderly men. The limitations of this study were as follows: first, primary and secondary SS were both included in this study; second, MPR was used to evaluate the drug adherence of HCQ, but irregular medication was usually found in SS patients; and third, most SS patients were female.

## 5. Conclusions

In summary, HCQ may be used to control the clinical manifestations of SS. HCQ has a wide range of positive effects including immunomodulation or immunosuppression. Significant cardiovascular protection of long-term HCQ therapy was observed from our findings. Therefore, adequate immunotherapy of HCQ is suggested in the patients with SS.

## Figures and Tables

**Table 1:** Clinical characteristics of the three-medication possession ratio (MPR) groups.

	Total		MPR < 0.40 (N = 653)		MPR = 0.40–0.69 (N = 215)		MPR ≥ 0.70 (N = 274)		
	*n*	%	*n*	%	*n*	%	*n*	%	*p*
Age									0.891
<40	302	26.4	178	27.3	51	23.7	73	26.6	
40–64	677	59.3	384	58.8	131	60.9	162	59.1	
≥65	163	14.3	91	13.9	33	15.3	39	14.2	
Mean ± SD	49.5 ± 14.5		49.4 ± 14.4		49.8 ± 15.3		49.5 ± 13.9		0.935
Gender									0.234
Female	997	87.3	562	86.1	188	87.4	247	90.1	
Male	145	12.7	91	13.9	27	12.6	27	9.9	
Hypertension	143	12.5	89	13.6	29	13.5	25	9.1	0.149
Hyperlipidemia	79	6.9	51	7.8	19	8.8	9	3.3	0.022
Diabetes	68	6.0	35	5.4	19	8.8	14	5.1	0.139
COPD	22	1.9	15	2.3	2	0.9	5	1.8	0.445
Stroke	33	2.9	16	2.5	5	2.3	12	4.4	0.239
Corticosteroids	451	39.5	159	28.8	104	48.4	188	58.0	<0.001
NSAIDs	787	68.9	434	66.5	149	69.3	204	74.5	0.056

MPR: medication possession ratio. COPD: chronic obstructive pulmonary disease. NSAIDs: non-steroidal anti-inflammatory drugs.

**Table 2:** The attack of coronary artery disease (CAD) in SS patients by different conditions.

	No. of CAD	Observed Person-Years	Incidence Density (Per 1000 Person-Years)	Crude HR	95% C.I.	Adjusted HR ^†^	95% C.I.
MPR of HCQ							
<0.40	60	653	91.9	1		1	
0.40–0.69	16	215	74.4	0.88	0.51–1.53	0.93	0.53–1.63
≥0.70	12	274	43.8	0.46	0.25–0.86	0.49	0.26–0.94
Age							
<40	8	1680	4.8	1		1	
40–64	53	3270	16.2	3.37	1.60–7.10	2.99	1.41–6.35
≥65	27	624	43.3	8.93	4.04–19.7	4.69	1.99–11.03
Gender							
Female	72	4850	14.8	1		1	
Male	16	723	22.1	1.49	0.87–2.57	1.15	0.65–2.05
Hypertension							
No	65	5071	12.8	1		1	
Yes	23	502	45.8	3.49	2.16–5.65	1.98	1.17–3.36
Hyperlipidemia							
No	74	5331	13.9	1		1	
Yes	14	242	57.8	3.96	2.22–7.06	2.27	1.18–4.38
Diabetes							
No	78	5327	14.6	1		1	
Yes	10	246	40.6	2.70	1.39–5.22	1.41	0.69–2.89
COPD							
No	81	5470	14.8	1		1	
Yes	7	103	68.0	4.70	2.17–10.18	3.57	1.51–8.46
Stroke							
No	83	5447	15.2	1		1	
Yes	5	127	39.4	2.48	0.78–6.13	1.17	0.44–3.10
Corticosteroids							
No	43	2926	14.7	1		1	
Yes	45	2647	17.0	1.18	0.78–1.80	1.23	0.79–1.93
NSAIDs							
No	10	831	12.0	1		1	
Yes	78	4742	16.4	1.47	0.75–2.86	1.36	0.68–2.70

MPR: medication possession ratio. Bold font represents statistical significance (*p* < 0.05). COPD: chronic obstructive pulmonary disease. NSAIDs: non-steroidal anti-inflammatory drugs. ^†^ Adjusted for age, gender, hypertension, hyperlipidemia, diabetes, COPD, stroke, corticosteroids and NSAIDs.

**Table 3:** The evaluation of CAD attack in patients with SS by different age and gender.

	N	No. of CAD	Crude HR	95% C.I.	Adjusted HR ^†^	95% C.I.
Age <40 ^†^						
MPR of HCQ						
<0.40	178	7	1		1	
0.40–0.69	51	1	0.48	0.06–3.88	0.40	0.05–3.49
≥0.70	73	0	NA	NA	NA	NA
Age = 40–64 ^†^						
MPR of HCQ						
<0.40	384	39	1		1	
0.40–0.69	131	8	0.65	0.31–1.40	0.58	0.27–1.25
≥0.70	162	6	0.34	0.14–0.79	0.28	0.12–0.68
Age ≥ 65 ^†^						
MPR of HCQ						
<0.40	91	14	1		1	
0.40–0.69	33	7	1.67	0.67–4.15	1.84	0.71–4.73
≥0.70	39	6	1.37	0.52–3.59	1.46	0.52–4.13
Female ^‡^						
MPR of HCQ						
<0.40	562	49	1		1	
0.40–0.69	188	15	1.03	0.58–1.84	0.99	0.55–1.80
≥0.70	247	8	0.37	0.17–0.77	0.36	0.16–0.77
Male ^‡^						
MPR of HCQ						
<0.40	91	11	1		1	
0.40–0.69	27	1	0.24	0.03–1.91	0.30	0.03–2.80
≥0.70	27	4	1.06	0.34–3.36	0.85	0.22–3.20

NA: not applicable. MPR: medication possession ratio. ^†^ Adjusted for gender, diabetes, corticosteroids and NSAIDs. ^‡^ Adjusted for age, hypertension, hyperlipidemia, diabetes, COPD, stroke, corticosteroids and NSAIDs.
